# Ethical dilemmas and legal ambiguity in China: a chain mediation model linking suicide rumination, legitimization, and acceptance among acutely-ill adults

**DOI:** 10.3389/fpsyg.2023.1342798

**Published:** 2024-01-30

**Authors:** Guo Liu, Kai Liu

**Affiliations:** ^1^School of Law, China University of Political Science and Law, Beijing, China; ^2^Health Law Research Center, China University of Political Science and Law, Beijing, China; ^3^Retirement Office, China University of Geosciences, Beijing, China; ^4^Mental Health Counseling Center, China University of Geosciences, Beijing, China

**Keywords:** bioethics, Chinese adults, cognitive depression, euthanasia, cultural attitudes, psychological aspects of suicide, suicide, terminally-ill patients

## Abstract

**Background:**

This study explores the complex intersection of euthanasia, legal ambiguities, cultural attitudes, and the psychology of suicide among seriously ill patients in China. It addresses the lack of clear legislation on euthanasia and doctor-assisted killing, the impact of cultural and philosophical beliefs, and the evolution of legal and ethical perspectives on suicide. Additionally, it examines the psychological aspects of suicide ideation in acutely-ill patients, focusing on factors like familial burden and loss of dignity.

**Method:**

A survey was conducted with 356 Chinese adults, aged 23 to 64 years, using popular social media platforms in China. The study aimed to reflect a broad spectrum of the adult population in terms of age, education, and professional sectors. The research model involves suicide rumination as an independent variable, acutely-ill patients' suicide acceptance as a dependent variable, and three mediators: cognitive depression, ethical suicide acceptance, and suicide legitimization.

**Results:**

The findings reveal a significant total effect of Suicide Rumination on Acutely-ill Patients' Suicide Acceptance, underlining a robust direct relationship that supports Hypothesis 1. The analysis indicates that Suicide Rumination is a significant predictor of Cognitive depression, explaining approximately 8.05% of its variance, thereby fully supporting Hypothesis 2a. However, the effect of Suicide Rumination on Ethical Suicide Acceptance did not emerge as significant, failing to support Hypothesis 2b, while its impact on Suicide Legitimization was also non-significant, not supporting Hypothesis 2c. Cognitive depression was found to have a substantial effect in the models for both Ethical Suicide Acceptance and Suicide Legitimization, supporting Hypotheses 3a and 3b. In the comprehensive model assessing Acutely-ill Patients' Suicide Acceptance, incorporating all mediators, a significant variance (R-squared = 0.6625) was explained. Notably, Suicide Rumination, Ethical Suicide Acceptance, and Suicide Legitimization all emerged as significant predictors of this acceptance, with varying effects, thus supporting Hypotheses 4a and 4b. The role of Cognitive depression in this model was marginally significant, offering limited support for Hypothesis 4c. Crucially, the indirect effects of Suicide Rumination on Acutely-ill Patients' Suicide Acceptance through different mediational paths varied in significance and impact. The indirect effect via Cognitive depression alone, and through the sequential combination of Cognitive depression and Ethical Suicide Acceptance, were significant, highlighting the nuanced role of these mediators. These findings underscore the importance of considering multiple pathways in understanding the dynamics of Suicide Rumination and its influence on the acceptance of suicide among acutely-ill patients.

**Conclusion:**

This paper presents a comprehensive analysis of the legal, cultural, ethical, and psychological dimensions of euthanasia, doctor-assisted killing, and suicide in China. The findings highlight the significant direct and mediated effects of suicide rumination on the acceptance of suicide among acutely-ill patients. The study contributes valuable insights into the evolving bioethics and the interplay of various factors in the context of end-of-life decision-making in modern Chinese society.

## Introduction

Euthanasia's intersection with legal, cultural, and ethical realms is increasingly significant in China (Yueqi, 2023). This paper delves into these complexities, focusing on the legality of euthanasia and doctor-assisted killing, attitudes toward different types of euthanasia, and the psychology of suicide among seriously ill patients.

In China, euthanasia and doctor-assisted killing present legal challenges. There is no clear law, creating a gray area where cultural and philosophical beliefs, deeply rooted in Confucianism, Buddhism, and Taoism, clash with practice (Li, 2023). This legal ambiguity reflects the cultural and ethical complexities, shaping opinions among healthcare professionals and the public. These attitudes vary, influenced by a range of beliefs and ethical considerations (Chong and Fok, 2009).

The paper also examines the legal and ethical views on suicide in China. Once morally frowned upon and influenced by Confucianism, the legal stance has shifted toward prevention and support, reflecting a deeper understanding of the complexities of suicide, mental health, and socio-economic factors (Zhang et al., 2002).

The differentiation between positive and negative euthanasia adds to this narrative. Positive euthanasia faces opposition due to ethical concerns and the sanctity of life principle, while negative euthanasia receives more nuanced responses, influenced by cultural values and global bioethical discussions. The legal uncertainty around both forms affects end-of-life care decision-making (Lei et al., 2022).

Additionally, the psychological aspects of suicide thoughts and acceptance in acutely-ill patients show the interplay between mental health, cultural context, and personal suffering. Factors like perceived familial burden, loss of dignity, and psychological distress impact suicidal ideation in terminally ill patients (Ibrahim and Amit, 2014).

This paper provides a comprehensive analysis of these facets, intertwining legal, cultural, ethical, and psychological perspectives in the context of euthanasia, doctor-assisted killing, and suicide in China. It contributes to the broader academic conversation on these vital issues, offering insights into the complexities and evolving bioethics in modern Chinese society.

## Theoretical framework

### Legality of euthanasia and doctor-assisted killing in China

In exploring the legality and ethical considerations of euthanasia and doctor-assisted killing in China, it is essential to consider the intertwining of legal, cultural, and ethical dimensions. The legal landscape in China regarding euthanasia remains undefined, primarily due to the lack of explicit legislation. In this context, euthanasia often falls into a legal gray area, with active euthanasia potentially treated as equivalent to homicide, and passive euthanasia left ambiguous (Ho, 1998).

Cultural and philosophical influences play a significant role in shaping attitudes toward euthanasia in China. The influence of Confucianism, which emphasizes filial piety and the sanctity of life, often leads to a general opposition to euthanasia, viewing it as contrary to the natural order and family obligations (Chen, 2019). Additionally, the perspectives from Buddhist and Taoist philosophies, which value life's sanctity and have complex views on suffering and death, further complicate the public and professional stances on euthanasia.

The attitudes of healthcare professionals and the public toward euthanasia in China are diverse. Studies have shown a range of opinions among healthcare professionals, with some showing cautious support under strict conditions, while others oppose it on ethical grounds (Ming-lin Chong and Fok, 2005). The general public's attitudes also reflect this divide, with some advocating for the right to die with dignity, especially in terminal illness cases, and others opposing it due to moral or cultural beliefs.

The academic debate in China around euthanasia and doctor-assisted killing calls for a comprehensive and nuanced approach. The need for clear guidelines that balance ethical considerations with practical needs is emphasized, particularly concerning the protection of vulnerable groups and the prevention of potential abuses (Nie, 2002; Chiesi et al., 2020). This debate highlights the complexity of the issue and the necessity of considering the ethical, legal, and cultural dimensions comprehensively.

In summary, the discussion on euthanasia and doctor-assisted killing in China is marked by an absence of specific legislation, deeply influenced by cultural and philosophical beliefs, and characterized by a spectrum of opinions among healthcare professionals and the public. The scholarly discourse emphasizes the need for a thoughtful and balanced approach to address this complex and sensitive issue.

### Bioethics: China's legal and ethical views on suicide

From a legal standpoint, the perspective on suicide in China has undergone significant transformation in recent years (Wu and Chassang, 2020). The Chinese legal system does not criminalize the act of suicide. This stance is a departure from historical views where, in certain periods, suicide could attract legal scrutiny, especially if it was connected to political or social dissent (Lee and Kleinman, 2000). In contemporary China, the approach is more aligned with public health and psychological welfare, focusing on prevention and support rather than legal consequences (Wang et al., 2022).

Ethically, the Chinese perspective on suicide is deeply influenced by cultural and societal values. Traditionally, Chinese society, under the influence of Confucian and Buddhist doctrines, has viewed suicide as morally problematic. Confucianism, in particular, emphasizes the importance of societal and familial duties, portraying suicide as an act of abandonment of these responsibilities (Chen et al., 2016; Wang and Zhang, 2023). However, in certain historical contexts, suicide has been seen as an honorable way to preserve personal or family dignity in the face of insurmountable shame or dishonor (Phillips and Liu, 1999).

In more recent times, there has been a shift in attitudes toward a more empathetic understanding of the complexities surrounding suicide. This shift is partly due to increased awareness of mental health issues and the impact of rapid socio-economic changes in Chinese society (Willemsen et al., 2021). The government and health agencies are increasingly focusing on mental health promotion, suicide prevention, and intervention strategies, recognizing the multi-faceted nature of suicide (Zhang et al., 2002). Empirical research has also been focused on suicide among specific populations, as elders (Cheng et al., 2014), cancer (Nigussie et al., 2023), mental health (Dong et al., 2019) or terminally – ill patients (Lam, 2007), survivors of child abuse (Liu et al., 2022), and subpopulations under specifically risks, as LGTBI+ youth (Wang et al., 2021), left-behind children (Xiao et al., 2019) or adolescents (Lai Kwok and Shek, 2010).

In conclusion, the legal and ethical views on suicide in China are characterized by an evolution from traditional cultural condemnations toward a more nuanced understanding that incorporates public health perspectives. This shift reflects broader changes in societal attitudes toward mental health and recognizes the complex interplay of cultural, social, and individual factors in the phenomenon of suicide.

### Attitudes toward positive and negative euthanasia in China

In examining Chinese attitudes toward positive and negative euthanasia, it is crucial to consider empirical studies that have explored these perspectives in various contexts, ranging from medical practitioners to the general public (Ming-lin Chong and Fok, 2005).

Positive euthanasia, often referred to as active euthanasia, involves direct actions to end a patient's life, such as administering lethal substances. In China, this form of euthanasia remains a controversial and largely disapproved practice. Some studies involving healthcare professionals in China revealed significant ethical concerns and opposition to positive euthanasia (Chong and Fok, 2009). The primary reasons cited were moral convictions and the sanctity of life principle, deeply rooted in Chinese culture and Confucian ethics (Yun et al., 2018).

Similarly, surveys conducted among the general public indicated a low level of acceptance for positive euthanasia. The majority of respondents expressed discomfort with the idea of actively ending a life, reflecting traditional views on the importance of natural death and the moral duty to preserve life (Lee et al., 2007; Lei et al., 2022).

Negative euthanasia, or passive euthanasia, involves withholding or withdrawing life-sustaining treatments. In contrast to positive euthanasia, negative euthanasia has a somewhat more nuanced reception in China. Research highlighted that while there is still considerable hesitation, a larger proportion of medical professionals in China are open to negative euthanasia, especially in cases where continued treatment is futile (Weng et al., 2011).

Studies focusing on end-of-life care in Chinese hospitals found that decisions regarding negative euthanasia are often influenced by family members, in line with the Confucian emphasis on family-oriented decision-making. This study also underscored the ethical dilemmas faced by healthcare providers in balancing respect for patient autonomy with traditional values (Hahne et al., 2022).

Public opinion on euthanasia in China is still evolving. Studies on the legal control of Euthanasia in China indicated a growing openness toward both forms of euthanasia, particularly among younger, more educated demographics. This shift is attributed to increased exposure to global ethical debates and a more profound understanding of patient suffering and dignity (Yueqi, 2023).

However, the legal framework in China does not currently recognize either form of euthanasia. The lack of legal clarity further complicates the ethical landscape, leaving healthcare providers and families in difficult positions when making end-of-life decisions (Lei et al., 2022). In summary, attitudes toward positive and negative euthanasia in China are complex and evolving. While there remains a predominant cultural and ethical opposition to positive euthanasia, there is a more nuanced approach to negative euthanasia, influenced by traditional values, family dynamics, and emerging global bioethical discourse.

### Suicide rumination and acutely-ill patients' suicide acceptance

Suicide rumination has been largely studied among younger populations, as college students, (Zou et al., 2023), athletes infected with COVID-19 (Zhao et al., 2023), somatic – anxiety patients (Sun et al., 2023), University students with depression (Sit et al., 2022) or adolescents with communication problems (Yan et al., 2020).

Suicide rumination, characterized as persistent contemplation about suicide, exhibits a notably high prevalence among acutely-ill patients. This prevalence necessitates a thorough examination of the psychological underpinnings and empirical research addressing the mental states and societal influences impacting individuals considering suicide (Giorgi et al., 2020). Underscore the importance of understanding these psychological dimensions, which not only reflect the individual's mental state but also the broader societal and cultural contexts.

Research indicates that acutely-ill patients, particularly those with terminal illnesses, often experience elevated levels of depression and hopelessness, contributing to increased suicidal ideation. The work of Cheung et al. (2006) highlights the critical role of psychological support in mitigating these thoughts, emphasizing the interconnectedness of mental health and suicidal tendencies in this population.

The cultural influence on suicide rumination is especially pronounced in Eastern contexts, as evidenced by the research of Joshi et al. (2017). Their findings suggest that the perception of being a burden to the family and a loss of dignity, concepts deeply rooted in Confucian values of familial duty and honor, significantly influence acutely-ill patients' contemplation of suicide. This cultural perspective provides an essential dimension to understanding the psychological experience of these patients.

Furthermore, the acceptance of suicide among acutely-ill patients emerges as a multifaceted psychological phenomenon. Leung et al. (2013) identified a correlation between the intensity of physical pain, psychological distress, and the inclination toward accepting suicide as a means to alleviate suffering. This relationship underscores the complexity of the decision-making process for these patients. Hu et al. (2023) contribute to this discussion by exploring the narratives of acutely-ill patients contemplating suicide. Their study reveals that factors such as loss of autonomy, fear of being a burden, and the desire to control the manner of death profoundly influence patients' acceptance of suicide. This acceptance, however, is not straightforward but rather a conflicted state, oscillating between the desire for life and the longing for relief from suffering.

In light of these findings, the significance of tailored mental health interventions for acutely-ill patients becomes evident. Duko and Gebeyehu (2015) argue that such interventions, addressing the specific psychological needs of these patients, are pivotal in managing suicidal ideation and enhancing their quality of life.

In summary, the phenomena of suicide rumination and acceptance among acutely-ill patients are intricately shaped by psychological distress, cultural values, and individual experiences of illness. The accumulated evidence underscores the imperative for comprehensive psychological and palliative care approaches that holistically address both the mental and physical aspects of patient well-being.

Based on the above revised literature, in the present study the following hypothesis is proposed:

Hypothesis 1: Suicide rumination (X) predicts Acutely-ill patients' suicide acceptance (Y).

### Mediating roles of cognitive depression, ethical suicide acceptance and suicide legitimization

The complex interplay between cognitive depression, the legitimization of suicide, and the ethical acceptance of suicide forms a pivotal mediating pathway in the transition from suicide rumination to its acceptance in acutely-ill patients, as proposed by Dong et al. (2019). This literature review aims to delve deeper into these mediating roles to enhance our understanding of their collective impact on the psychological state of these patients.

Cognitive depression, characterized by distorted thinking patterns, negative self-perception, and a pervasive sense of hopelessness, significantly influences patients' cognitive and emotional states. In the context of chronic or terminal illness, cognitive depression can exacerbate feelings of helplessness and existential distress. The study by Cheng et al. (2014) underscores this phenomenon, illustrating how cognitive depression can intensify suicidal ideation among chronically ill patients. This correlation is paramount, as it suggests that the mental state of the patient significantly influences their perception and rationalization of suicide.

The concept of suicide legitimization emerges as a crucial factor in this milieu. Research conducted by Zhong et al. (2017) sheds light on how societal and cultural attitudes can shape an individual's perception of suicide. In certain cultural contexts, where enduring pain and suffering is sometimes valorized, suicide can be perceived differently – as a rational and dignified escape from unbearable suffering. This societal perspective is instrumental in legitimizing the act of suicide for patients suffering from severe cognitive depression.

The interconnection between cognitive depression and the legitimization of suicide is a critical one. Cognitive depression does not only heighten the frequency and intensity of suicidal thoughts but also fundamentally alters the patients' perception of these thoughts. In the depths of severe depression, the notion of suicide can evolve from a taboo or intimidating concept to a legitimate, rational option. This transformation becomes particularly significant in terminal illness, where the confluence of physical and emotional pain can be overwhelming. The cumulative effect of cognitive depression, combined with the process of suicide legitimization, culminates in an increased acceptance of suicide. This acceptance signifies a shift in the patients' perspective, where suicide is viewed as a conceivable and ethically justifiable option, as indicated by Park et al. (2016). This perceptual shift is pivotal in understanding the psychological state of acutely-ill patients and highlights the imperative for comprehensive mental health support and ethical guidance in their care.

In summary, the literature reveals a nuanced and complex relationship between cognitive depression and the legitimization of suicide. This interplay is crucial in shaping the attitudes of acutely-ill patients toward suicide. Understanding and addressing these factors is essential in providing effective psychological and ethical support to these patients, potentially influencing their perspectives on life and death decisions.

This deeper exploration into the mediating roles of cognitive depression and suicide legitimization provides a more comprehensive understanding of the psychological processes underpinning suicide acceptance in acutely-ill patients. The objective of this study is to explore the chain mediating model of Suicide rumination and Cognitive depression, ethical suicide acceptance and suicide legitimization in predicting Acutely-ill patients' suicide acceptance among adults in China. Based on the above revised literature, in the present study the following hypotheses are proposed:

Hypothesis 2: Suicide rumination (X) predicts Cognitive depression (M1) (H2a), and Ethical suicide acceptance (M2) (H2b), and Suicide legitimization (M3) (H2c).
Hypothesis 3: Cognitive depression (M1) predicts Ethical suicide acceptance (M2) (H3a) and Suicide legitimization (M3) (H3b).
Hypothesis 4: Cognitive depression (M1) serves as a chain mediating variable that influences the relationship between the Suicide rumination (X) and two mediating variables (Ethical suicide acceptance. M2 and Suicide legitimization (M3), and the Acutely-ill patients' suicide acceptance (Y). In more detail, Cognitive depression (M1) exerts an influence on Ethical suicide acceptance and predicts the Acutely-ill patients' suicide acceptance (Y) (H 4a). Cognitive depression (M1) influences Suicide Legitimization (M3), and predicts the Acutely-ill patients' suicide acceptance (Y) (H 4b). Finally, Cognitive depression (M1) predicts the Acutely-ill patients' suicide acceptance (Y) (H 4c).

**Figure 1:**
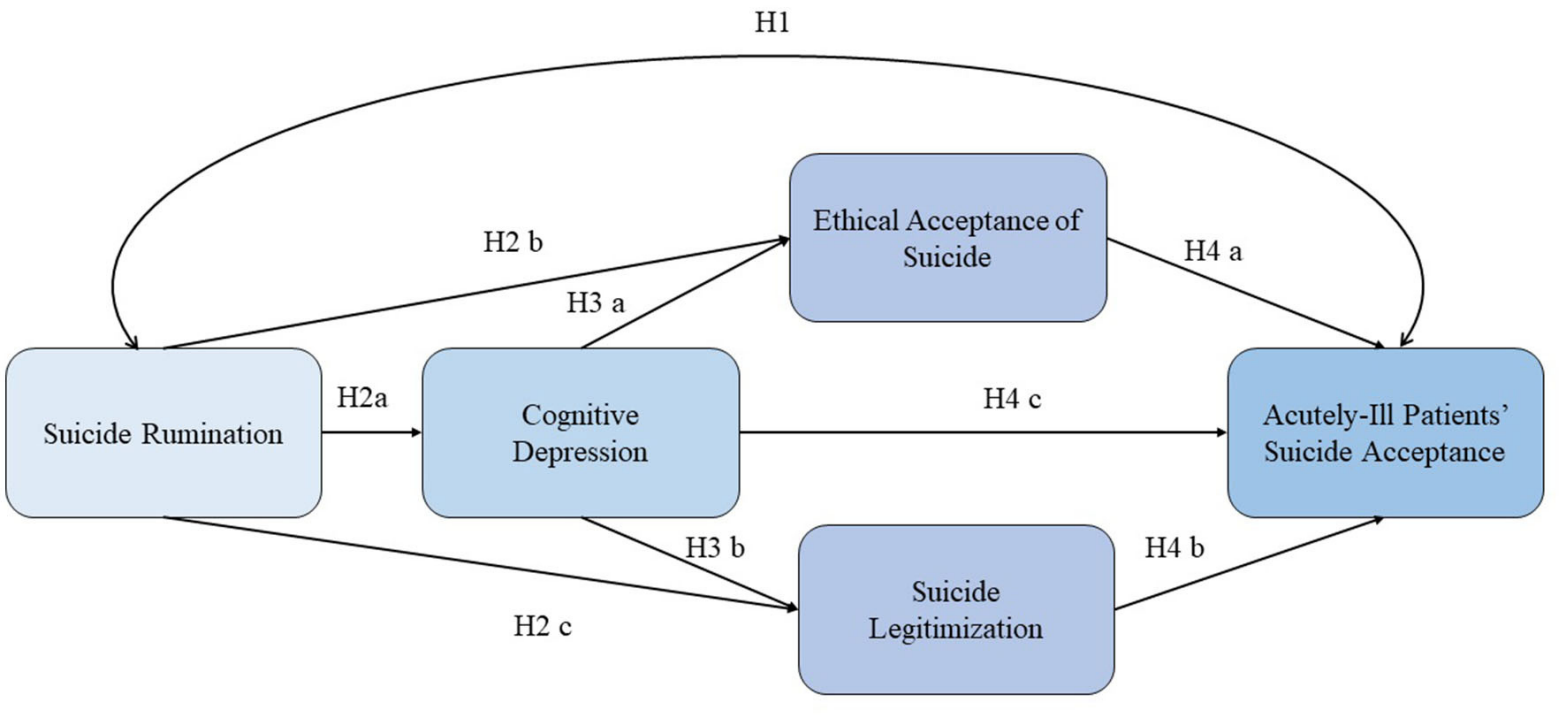
Research model and hypotheses.

The research model and the hypotheses are displayed in Figure 1.

## Method

### Participants and procedure

The study surveyed 356 Chinese adults, aged between 23 and 64 years, to understand a range of perspectives reflective of the broader adult population. This demographic composition was deliberately chosen to mirror the general adult populace in China, particularly in terms of age distribution (Mean age= 42.3; S. D. = 10.8). The aim was to ensure that the study's findings would be applicable and generalizable across different age groups within the adult population.

Data collection was executed using several popular social media platforms in China, namely Weibo, WeChat, and Douyin. These platforms were selected for their widespread use and diverse user bases, which are essential for reaching a broad spectrum of the population. By leveraging these platforms, the study was able to access a wide demographic, encompassing various educational, social, and economic backgrounds. Males were the 39% and 56.3% of the sample hold a Higher education degree, while 11.2% have Vocational training and 7.3% only primary studies. The majority of the sample was employed (70.8%), with only 7.2% as self-employed, 1.1 % retired and 10.9% still studying. Among those who work, Education (46.2%) and Services and Hospitality (27.6%) were the main professional sectors, followed by healthcare (10%) and Information Technology and Telecommunications (11.4%).

The survey was administered via a Qualtrics questionnaire, during the first semester of 2023. Renowned for its efficacy in online surveys. The choice of Qualtrics was driven by its compatibility with mobile devices, a crucial aspect considering the high prevalence of mobile internet usage in China. This mobile-friendly nature of the survey ensured that it was easily accessible, increasing the likelihood of higher participation rates and more varied responses. Despite that the final collection provided responses from 380 participants, those respondents that did not reach the 100% of the survey response where eliminated (*n* = 24).

The questionnaire was designed to be clear and concise, respecting the participants' time and ensuring ease of understanding for individuals from various educational backgrounds.

### Ethics statement

In February 2023, the National Science and Technology Ethics Committee of China approved the “Measures for Ethical Review of Life Science and Medical Research Involving Human Being,” which were jointly issued by the National Health Commission, the Ministry of Education, the Ministry of Science and Technology, and the State Administration of Traditional Chinese Medicine. According to Article 32, if anonymous information data is used to conduct life science and medical research involving human being, which does not cause harm to the human being, does not involve sensitive personal information or commercial interests, ethical review may be exempted to reduce unnecessary burden on researchers. This study is conducted using anonymous information data, which meets the above regulations and can be exempted from ethical review. During the study period, participants were provided with comprehensive information about the study's aims, procedures, and their role. Emphasizing voluntary participation, individuals were informed of their right to withdraw from the study at any time without consequences. The anonymity and confidentiality of responses were upheld rigorously throughout the study, aligning with data protection laws to safeguard participant privacy.

Informed consent was a prerequisite for participation. This consent was obtained digitally through the Qualtrics platform, ensuring that participants were fully aware of and agreed to the study terms before proceeding. Obtaining the consent of participants in this way complied with Article 14 of the Personal Information Protection Law of the People's Republic of China.

In summary, the entire study design, including the process of observing consent and managing data, fully complied with the relevant provisions of Chinese law.

### Instruments

*Suicide rumination*: This variable was assessed with the Suicide Rumination Scale (SRS) (Rogers et al., 2022), containing eight items that reflect a repetitive negative thought pattern that an individual might have regarding their suicidal thoughts. Participants were asked to indicate how often they typically experienced each thought/image when experiencing thoughts of suicide. Items were rated on a 5-point scale with response options including 0 (Almost never), 1 (Rarely), 2 (Sometimes), 3 (Often), and 4 (Almost always). The full items list is provided in Table 1. Despite the most common instrument to assess rumination is the Ruminative Response Scale, this is a general measure of rumination as a method of coping. Instead of this, in the present study, a suicide-specifically oriented rumination questionnaire was used. The items have been translated to Chinese independently by two of the authors, that are Chinese speaking and fluent in English. These translations were then evaluated and deliberated upon by both the experts and the research team to finalize the scale's version. To verify the accuracy of the translated scale, two separate back-translations into English were conducted. The research team examined any incongruities between this and the original Suicide Rumination Scale, resolving them through collective agreement, which led to the establishment of the final Chinese version of the SRS Scale, termed SRS-Chn (see Table 1). Reliability in the present study was 0.76.

**Table 1:** Suicide rumination scale (SRS) English and Chinese versions.

**Suicide rumination scale (SRS) English version**	**Suicide rumination scale (SRS-Chn) Chinese version**	**Mean**	**S. D**	**Skewness**	**Kurtosis**
When I have thoughts of suicide, I…	当我有自杀的念头时,我...	
1. Cannot “turn off” these thoughts	1. 无法 "关闭 "这些想法	3.722	1.373	−0.762	−0.711
2. Cannot escape these thoughts	2. 无法摆脱这些想法	3.413	1.396	−0.409	−1.127
3. Have trouble getting the suicidal thoughts out of my mind	3. 难以摆脱自杀的念头	3.096	1.373	−0.245	−1.155
4. Am unable to stop thinking about suicide	4. 无法停止自杀的念头	3.736	0.977	−0.979	1.266
5. Think about how I want to kill myself	5. 想着如何自杀	3.404	1.189	−0.560	−0.622
6. Imagine what killing myself with different methods would be like	6. 想象用不同的方法自杀会是什么样子	3.604	1.142	−0.628	−0.328
7. Wonder what the fastest and easiest way to die is	7. 想知道什么是最快最简单的死法	3.781	0.927	−0.725	0.775
8. Imagine the process of how I want to kill myself	8. 想象我想自杀的过程	3.213	1.219	−0.349	−0.752

Descriptive statistics of the items.

*Cognitive depression*: In the present study, the Cognitive component of the Zung's Self-Rating Depression Scale (SDS) (Zung, 1986) was used. This instrument has been applied in different cultures, as Japan (Sakamoto et al., 1998), and Colombia (Villacieros et al., 2016), as well as a tool discrimination for depression among cancer patients (Dugan et al., 1998) or elderly Chinese participants (Dugan et al., 1998), being useful and reliable (Thurber and Snow, 2002). Like previous studies, in the present research, only the cognitive component of the SDS has been used. Cognitive processes refer to the methods employed by humans to acquire knowledge, involving capabilities such as intelligence, attention, memory, and language. The original scale's Cronbach alpha ranged from 0.88 to 0.93. Our sample showed a Cronbach's alpha = 0.78. An example of the items is “I still enjoy the things I used to do.”

*Ethical suicide acceptance:* this variable has been assessed with four items from the Suicide Acceptance Questionnaire (Stecz and Gmitrowicz, 2020). It is comprised of the items that showed higher loadings into the one factor solution of the instrument, as shown in Table 2. The internal consistency of the dimension in the original study was 0.92.

**Table 2:** Descriptive statistics of items for the suicide legitimization, ethical suicide acceptance, and support toward suicidal people subscales.

**Ethical suicide acceptance subscale English version**	**Ethical suicide acceptance subscale Chinese version**	**Mean**	**S. D**	**Skewness**	**Kurtosis**
Bankruptcy justifies suicide	破产证明自杀合理	4.678	1.594	−1.038	−0.128
Love failure can justify suicide	爱情失败可以成为自杀的理由	4.793	1.541	−0.979	−0.425
Feeling of helpless and hopelessness are acceptable reasons for suicide	感到无助和绝望是可以接受的自杀理由	5.133	1.220	−1.239	0.586
Life sentence may be reasonable to commit suicide	无期徒刑可能是自杀的合理理由	5.546	0.724	−1.803	3.255
***Suicide legitimization subscale English version***	***Suicide legitimization subscale Chinese version***				
1. I would accept certain forms of suicide (e.g., pill overdose).	我接受某些形式的自杀（如服药过量）	3.469	2.203	0.138	−1.556
2. I would accept suicide in the elderly	我可以接受老人自杀	3.666	2.190	0.147	−1.462
3. There should be clinics so that suicidal people can take their own lives in a private way and with less suffering.	应该设立一些诊所,让有自杀倾向的人能够私下结束自己的生命,减少痛苦	3.733	2.171	0.212	−1.378
4. If someone wants to attempt suicide, it is their own business, and we should not intervene.	如果有人想自杀,那是他们自己的事,我们不应干涉	2.298	1.557	1.095	0.394
5. Suicide should be a legitimate way of dying	自杀应该是一种合法的死亡方式。	4.772	2.127	−0.521	−1.178
6. Suicide would be a normal thing to do in an ideal society.	在理想社会中,自杀是一件正常的事情。	2.795	1.891	0.705	−0.760
***Support toward suicidal people subscale English version***	***Support toward suicidal people subscale Chinese version***				
Most people are willing to offer psychological support to acutely ill patients who want to attempt or commit suicide.	大多数人愿意为企图自杀或自杀的重病患者提供心理支持。	5.688	1.598	−1.272	1.065
Most people are willing to offer material support to acutely ill patients who want to attempt or commit suicide.	大多数人愿意为企图自杀或自杀的急性病患者提供物质支持。	4.525	2.112	−0.399	−1.276
Most people care about those acutely ill patients that had suicidal attempts.	大多数人关心企图自杀的急性病患者。	5.185	1.881	−0.840	−0.374
Acutely ill patients can solve problems that may lead to suicide through personal effort. (^*^).	急性病患者可以通过个人努力解决可能导致自杀的问题。	5.146	1.865	−1.024	0.044

^*^Reversed scored.

*Suicide legitimization:* this variable has been assessed with the Suicide Legitimization dimension of the Suicidal Behavior Beliefs Questionnaire (CCCS-18) (Hernández et al., 2005). The scale includes six items pertaining to the perception of suicide as rationally acceptable. The list of items is included in Table 2. The internal consistency of the dimension in the original study 0.88, and its psychometric properties have been later confirmed in other studies (Desuque and Rubilar, 2011).

*Acutely-Ill patients' suicide acceptance*: for the assessment of this variable, we used the: support toward suicidal people subscale of the Chinese attitude toward suicide questionnaire (CASQ) (Lee et al., 2007). The measure includes the four items that showed higher loading into the factor, and the redaction has been adapted to relate the item to suicide in terminally ill patients who have no chance of recovery. The more general word “friends” has been replaced by “terminally ill patients.” The internal consistency of the dimension in the original study was 0.61, but in the present study it was 0.90.

The full list of items for the questionnaires is displayed in Table 2.

### Data analyses

The analysis of the data was conducted using the Statistical Package for the Social Sciences (SPSS) version 24 (IBM Corp, 2016). The PROCESS 4.2 macro (Hayes, 2022) facilitated the examination of chain mediation. The initial stage involved determining the relationships between various variables via Pearson's correlation. Subsequently, a chain mediational analysis was performed employing Model 81. This model tested the chain mediation where Cognitive depression mediating role was analyzed in the context of the relationship between Suicide Rumination and Acutely-ill patients' suicide acceptation. This mediation was further influenced by prior influence of Cognitive depression on Ethical Suicide Acceptance, and Suicide legitimization. The support for the chain mediation hypothesis is contingent upon the exclusion of zero from the 95% bias-corrected confidence interval. Should this condition be met, it can be inferred that the parameter in question significantly deviates from zero at *p* < 0.05. The analysis was underpinned by 5,000 bootstrap re-samples and included estimates of the indirect effects. The sample of analysis for the descriptive statistics and Pearson's correlation was 356 participants, but PROCESS eliminated those respondents with at least one missing data, taking into account only 350 for the mediational analyses.

## Results

### Descriptive statistics and Pearson's correlation matrix

In examining Table 3, we observe significant correlations between the studied variables, which are indicative of the complex relationships inherent in our research focus. The table illustrates the interplay between cognitive depression, the legitimization of suicide, and the ethical acceptance of suicide, highlighting how these variables interact in the context of acutely-ill patients.

**Table 3:** Pearson's Correlation matrix (*N* = 356).

**Variable**	**Mean**	**S. D**.	**1**	**2**	**3**	**4**
1. Suicide rumination	3.51	0.53	—			
2. Cognitive depression	2.95	1.47	0.284^***^	—		
3. Ethical suicide acceptance	5.01	1.03	0.059	0.297^***^	—	
4. Suicide legitimization	3.45	1.60	0.177^***^	0.612^***^	0.530^***^	—
5. Acutely-ill patients' suicide acceptance	5.13	1.63	0.301^***^	0.467^***^	0.574^***^	0.773^***^

^***^ p < 0.001.

The strength and direction of these correlations are noteworthy, as they provide insight into how these constructs are interrelated. For instance, the positive correlations suggest that as cognitive depression intensifies, there tends to be a corresponding increase in the legitimization of suicide and its ethical acceptance. This finding is consistent with the theoretical underpinnings of our study, reinforcing the notion that cognitive depression can be a pivotal factor in shaping patients' attitudes toward suicide.

Moreover, the results underscore the nuanced nature of these relationships. The varying degrees of correlation between different pairs of variables suggest that while there is a general trend, the influence of each factor on the others is complex and multifaceted. This complexity is a critical aspect of our findings, as it highlights the need for a holistic approach in understanding and addressing the psychological state of acutely-ill patients.

### Mediating model

We used the PROCESS Procedure for SPSS, developed by Hayes (2022), for testing the model involving one independent variable Suicide Rumination, one dependent variable Acutely-ill patients' suicide acceptance, and three mediator variables cognitive depression, ethical suicide acceptance, suicide legitimization, with 350 participants without missing data.

The analysis of total, direct, and indirect effects highlights the complex interplay between these variables: the total effect of suicide rumination on acutely-ill patients' suicide acceptance is significant, while the direct effect of suicide rumination on acutely-ill patients' suicide acceptance, when controlling for mediators, remains significant, as it is displayed in Table 4.

**Table 4:** Unstandardized values of variables in the model.

	**Coeff**.	**SE**	***t***	***p***	**LLCI**	**ULCI**
**Outcome variable: suicide legitimization**						
Constant	1.5183	0.4517	3.3615	0.0009	0.6299	2.4066
Suicide Rumination	0.0152	0.1322	0.1148	0.9086	−0.2448	0.2752
Cognitive depression	0.6441	0.0484	13.3181	0.0000	0.5490	0.7392
	**Coeff**.	**SE**	***t***	***p***	**LLCI**	**ULCI**
**Outcome variable: acutely-ill patients' suicide acceptance**						
**Model**						
Constant	−0.6662	0.3959	−1.6827	0.0933	−1.4450	0.1125
Suicide rumination	0.5895	0.0946	6.2347	0.0000	0.4035	0.7755
Cognitive depression	−0.0804	0.0425	−1.8910	0.0595	−0.1640	0.0032
Ethical suicide acceptance	0.3623	0.0553	6.5540	0.0000	0.2535	0.4710
Suicide legitimization	0.6351	0.0432	14.6896	0.0000	0.5501	0.7201
	**Coeff**.	**SE**	***t***	***p***	**LLCI**	**ULCI**
**Total and direct effect model**						
**Outcome variable: acutely-ill patients' suicide acceptance**						
Constant	2.0540	0.5261	3.9046	0.0001	1.0194	3.0887
Suicide rumination	0.8954	0.1477	6.0629	0.0000	0.6049	1.1859
**Effect**	**SE**	***t***	***p***	**LLCI**	**ULCI**	**c'_cs**
**Direct effect of X on Y**						
0.5895	0.0946	6.2347	0.0000	0.4035	0.7755	0.2035

The total effect model of Suicide Rumination on Acutely-ill patients' suicide acceptance (without mediators) reveals a significant effect, indicating a strong direct relationship, providing fully support for Hypothesis 1.

In the model assessing Cognitive depression as the outcome variable, there is a significant association with Suicide Rumination, as indicated by an R-squared value of 0.0805. Specifically, suicide rumination positively predicts cognitive depression. Suicide rumination explains about 8.05% of the variance in cognitive depression, fully supporting Hypothesis 2a.

The model with ethical suicide acceptance as the outcome variable, the R-squared value is 0.0891, with Suicide Rumination and Cognitive depression together explaining approximately 8.91% of the variance. Here, the effect of Suicide Rumination is not significant, failing to support H2b, but Cognitive depression shows a significant relationship, supporting H3a.

For Suicide legitimization as the outcome variable, the model is more robust, with an R-squared value of 0.3584. The effect of Suicide Rumination on Suicide legitimization is not significant, failing to support H2c. Cognitive depression has a significant and substantial effect, supporting H3b, while the effect of Suicide Rumination is not significant. Here, Cognitive depression and Ethical Suicide Acceptance significantly predict Suicide legitimization.

When examining Acutely-ill patients' suicide acceptance as the outcome variable, the model including all mediators explains a significant proportion of variance (R-squared = 0.6625). Suicide Rumination, Ethical Suicide Acceptance, and Suicide legitimization all significantly predict Acutely-ill patients' suicide acceptance, with Suicide Rumination having a positive effect. The effects of Cognitive depression and Ethical Suicide Acceptance are negative and positive, respectively, while Suicide legitimization shows a strong positive impact, fully supporting H4a, and H4b. All variables show a significant direct effect on the criterion, while for Cognitive depression the significance is only marginal, providing limited support for H4c.

**Figure 2:**
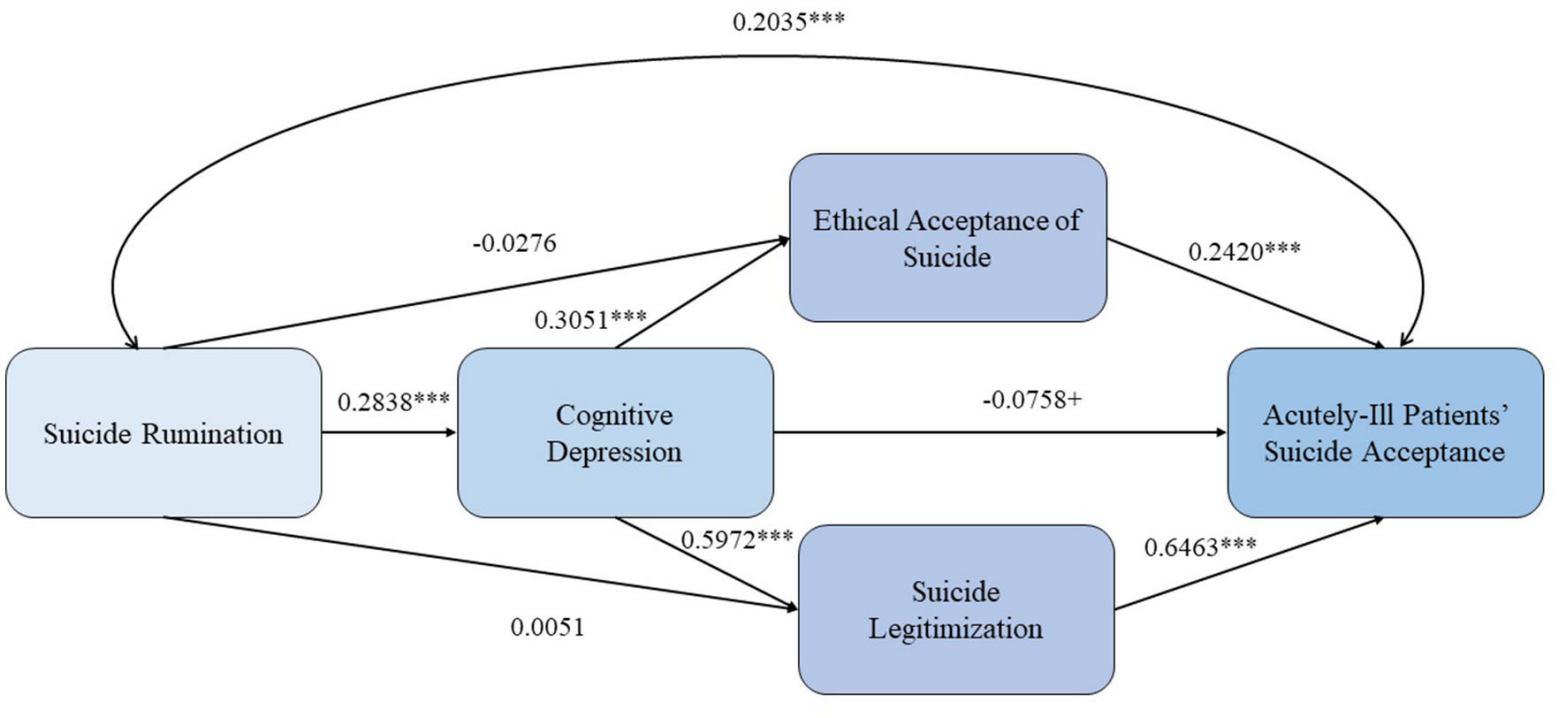
Standardized effects of the variables into the research model. ***p < 0.001; ^+^*p* < 0.10.

Turning into the indirect effects through various paths (see Table 5) vary in significance and strength, with some paths showing no significant indirect effect. The results suggest that while Suicide Rumination has a direct impact on Acutely-ill patients' suicide acceptance, its relationship is also mediated through different pathways, each contributing differently to the outcome. The significance of these paths highlights the importance of considering multiple mediators in understanding the influence of Suicide Rumination on Acutely-ill patients' suicide acceptance. In fact, the indirect effect 1 (Suicide Rumination->Cognitive depression->Acutely-ill patients' suicide acceptance), was significant, as well as the fourth (Suicide Rumination->Cognitive depression->Ethical Suicide Acceptance-> Acutely-ill patients' suicide acceptance), and the fifth (Suicide Rumination->Ethical Suicide Acceptance-> Suicide legitimization->Acutely-ill patients' suicide acceptance) showing the strong impact of cognitive depression on the outcome, providing support for Hypothesis 4. Moreover, both paths showed opposite signs, being the path through cognitive depression negative, and the fourth, positive. All the remaining paths were not significant. All the standardized effects are shown in Figure 2.

**Table 5:** Completely standardized indirect effects.

**Standardized indirect effects**	**Effect**	**Boot SE**	**Boot LLCI**	**Boot ULCI**
Total	0.1056	0.0424	0.0186	0.1857
Indirect 1: Suicide Rumination (X)->Cognitive depression (M1)->Acutely-ill patients' suicide acceptance (Y)	−0.0215	0.0118	−0.0477	−0.0014
Indirect 2: Suicide Rumination (X)->Ethical Suicide Acceptance (M2)->Acutely-ill patients' suicide acceptance (Y)	0.0143	0.0141	−0.0126	0.0429
Indirect 3: Suicide Rumination (X)->Suicide legitimization (M3)->Acutely-ill patients' suicide acceptance (Y)	0.0102	0.0260	−0.0406	0.0618
Indirect 4: Suicide Rumination (X)->Cognitive depression (M1)->Ethical Suicide Acceptance (M2)-> Acutely-ill patients' suicide acceptance (Y)	0.0879	0.0193	0.0514	0.1277
Indirect 5: Suicide Rumination (X)->Ethical Suicide Acceptance (M2)-> Suicide legitimization (M3)->Acutely-ill patients' suicide acceptance (Y)	0.0147	0.0141	−0.0142	0.0412

## Discussion

At the outset of our discussion, it is imperative to underscore the novel contributions of this study to the understanding of suicide-related phenomena. The current research distinctively elucidates the intricate relationships between suicide rumination, cognitive depression, ethical suicide acceptance, suicide legitimization, and the acceptance of suicide among acutely-ill patients. A major innovation of this study is the identification of a significant positive correlation between suicide rumination and both cognitive depression and suicide legitimization, as evidenced by our Pearson's correlation matrix. This correlation not only underscores the concurrent increase in these variables but also offers new insights into their interplay.

Additionally, our use of the PROCESS Procedure for SPSS in mediation analysis reveals the predictive influence of suicide rumination on cognitive depression, quantifying this relationship and thereby reinforcing existing literature, while also advancing our understanding of the ruminative thought patterns in depressive symptoms. The absence of a significant impact of suicide rumination on ethical suicide acceptance suggests a novel underlying mechanism in the formation of ethical views on suicide, a finding that diverges from conventional expectations.

Furthermore, this research innovatively highlights the mediating role of cognitive depression in the relationship between suicide rumination and suicide legitimization. This finding is particularly salient as it indicates that depressive symptoms are a critical factor in how individuals perceive the legitimacy of suicide, offering new perspectives for psychological and clinical interpretations. The robust model assessing suicide legitimization as an outcome, with a substantial variance explained by cognitive depression and ethical suicide acceptance, marks a significant advancement in this field.

Moreover, the study's examination of acutely-ill patients' acceptance of suicide through various mediated pathways sheds light on a complex interplay of factors, revealing the multifaceted nature of this acceptance. The indirect effects analysis further emphasizes the uniqueness of each pathway's contribution, a complexity that is both novel and of substantial practical relevance.

In summary, the findings of this study not only bridge gaps in existing research but also open new avenues for future investigations into the nuanced dynamics of suicide-related attitudes and behaviors. These contributions are crucial for a deeper understanding and more effective clinical management of suicide-related phenomena. The study notably supports Hypothesis 4, emphasizing the strong impact of cognitive depression on acutely-ill patients' acceptance of suicide. The path involving cognitive depression as a mediator is significant and negative, contrasting with the positive path through ethical suicide acceptance. This dichotomy underscores the nuanced nature of the relationship between mental health variables and attitudes toward suicide.

Our findings are in line with previous research in different ways. Recent research confirmed the association between depression and suicide risks (Rueda-Jaimes et al., 2018), as well as among rumination and suicidal ideation in later life (Liu et al., 2022). This evidence coincides with other studies that found influences of psychological strain and suicide among Chinese college students (Wang et al., 2020). In the same vein, relationships between predictors and suicidal ideation are shown complex and subjected to chain mediating relationships (Zheng et al., 2023).

The impact of suicidal attitudes on the Ethical Suicide acceptance and the Acutely-ill patients' suicide acceptance also coincides with previous findings, that showed suicidal ideation association with chronic disease prevalence, and suicide attempts as well as its effects on quality of life (Joshi et al., 2017). Consistently research showed that suicide precipitated by acute health problems is viewed as acceptable, as rational or courageous (Winterrowd and Canetto, 2017), enhancing its ethical acceptance. At the same time, other researchers showed discrepant findings, as individual attempts to reduce the ambiguity of suicide rumination or the cognitive dissonance mechanisms that moderate suicidal ideation and increase negative judgments toward suicide (Zhang et al., 2009).

In conclusion, our findings elucidate the intricate relationships between suicide rumination, cognitive depression, ethical considerations, and the acceptance of suicide among acutely-ill patients. The study highlights the crucial role of cognitive depression as a mediator, emphasizing the need for a multifaceted approach in understanding and addressing the complexities surrounding suicide acceptance and legitimization. The diverse impacts of these variables underscore the importance of considering multiple mediators in comprehensively understanding the dynamics of suicide rumination and its effects on suicide-related attitudes and behaviors.

### Limitations of the present study and suggestions for future research

This study, while comprehensive in its approach, presents several limitations that should be acknowledged. Firstly, the demographic profile of the participants, primarily Chinese adults aged between 23 years and 64 years, may limit the generalizability of the findings. Despite efforts to reflect the broader adult population of China, the specific age range and cultural context may not fully represent the diverse experiences and perspectives of adults in other regions or age groups (Wang and Zhang, 2023), as well as specific populations (Kuo, 2018).

Secondly, the use of social media platforms such as Weibo, WeChat, and Douyin for data collection, although effective in reaching a wide audience, might introduce selection bias. The nature of social media usage and the type of users active on these platforms could influence the study's findings, potentially skewing them toward a more technologically savvy and connected population (Lai et al., 2014).

The method of administering the survey, primarily through a mobile-friendly Qualtrics questionnaire, while beneficial for accessibility, may also limit participation from individuals less familiar or comfortable with mobile internet usage or online surveys. This could result in a sample that is not fully representative of the broader population, especially considering varying levels of technological access and literacy across different demographic groups.

Furthermore, the instruments used to measure the study variables, such as the Suicide Rumination Scale (SRS-Chn) and the Cognitive component of the Zung's Self-Rating Depression Scale (SDS), while reliable, have their own limitations. The translation and adaptation processes for these instruments, despite rigorous back-translation and evaluation, may not perfectly capture the nuances of the original scales. This could affect the validity of the measures and the accuracy of the findings (Ghasemi and Shaghaghi, 2015).

Future research should consider expanding the participant demographic to include a broader age range and cultural backgrounds (Garcia-Fernández et al., 2021), to enhance the generalizability of the results (Renger et al., 2023). Additionally, employing a variety of data collection methods beyond social media platforms can help mitigate selection bias and ensure a more representative sample. Further validation studies of the translated instruments are recommended to confirm their reliability and accuracy in different cultural contexts. Exploring additional variables that might influence suicide rumination and depression, such as socioeconomic factors or access to mental health services, would also provide a more comprehensive understanding of these complex issues.

### Intervention and practitioner's recommendations in mental health

In addressing the issues of suicide rumination and cognitive depression, as highlighted in recent studies, several key recommendations for intervention and practice in the field of mental health are pertinent. These suggestions aim to enhance the effectiveness of mental health services and reduce the prevalence and severity of these conditions.

Firstly, there is a critical need for culturally sensitive approaches in mental health interventions. The variations in how cognitive depression and suicide are perceived and experienced across different cultures necessitate tailored approaches. Mental health practitioners should be trained in cultural competency, enabling them to recognize and respect the unique cultural backgrounds and beliefs of their clients. This training should include an understanding of cultural stigmas associated with mental health, which can significantly impact help-seeking behavior and treatment acceptance (Liu et al., 2019).

Secondly, increasing accessibility to mental health services is paramount. This includes expanding services in underserved areas, offering flexible hours for appointments, and providing options for tele-therapy or online counseling. Such measures can help reach individuals who might otherwise face barriers to accessing care, such as those living in remote areas, having mobility issues, or experiencing scheduling conflicts.

Furthermore, integrating mental health education into public health initiatives can play a significant role in destigmatizing mental health issues. Educational campaigns should focus on raising awareness about the signs and symptoms of cognitive depression and suicide risk, encouraging early intervention and treatment. These campaigns can be disseminated through various media channels and public forums, reaching a broad audience.

Practitioners should also prioritize the development of individualized treatment plans, acknowledging that each person's experience with cognitive depression and suicidal thoughts is unique (Anglin and Gabriel, 2005). Personalized treatment may involve a combination of therapeutic approaches, such as cognitive-behavioral therapy, medication, and lifestyle interventions. Regular assessments and adjustments to treatment plans are crucial to ensure they remain effective and responsive to the client's needs (Wong, 2018).

In addition, there is a need for ongoing research and training for mental health professionals. This includes staying updated with the latest evidence-based practices and emerging trends in mental health care (Yousuf et al., 2013). Practitioners should engage in continuous professional development to refine their skills and knowledge, ensuring that they provide the highest standard of care to their clients.

In light of these recommendations for mental health interventions and practices, it is equally important to consider the broader societal implications and policy responses that stem from our study's findings. The integration of these considerations with targeted mental health interventions can lead to a more holistic approach to addressing suicide rumination and cognitive depression, ultimately benefiting individuals and society alike.

The societal and policy implications of our findings suggest that interventions at the community and policy level are also crucial. Educators, policymakers, and community leaders should be informed about the complexities and interrelationships of suicide rumination, cognitive depression, and suicide legitimization, as revealed by our study. By understanding these dynamics, they can formulate more effective policies and educational programs that address the root causes and contributing factors of these mental health issues. At the policy level, there should be a focus on creating supportive environments that reduce the stigma around mental health and provide accessible resources for those in need. This includes funding for mental health programs, support for research in the field, and the development of policies that protect the rights and well-being of individuals with mental health challenges.

In summary, our study not only contributes to the understanding of specific mental health issues but also underscores the importance of comprehensive approaches that encompass clinical interventions, community education, and supportive policies. By addressing these issues at multiple levels, we can better support individuals affected by cognitive depression and suicide rumination, and make strides toward improving mental health outcomes on a societal scale.

## Conclusion

This paper has meticulously explored the multifaceted dynamics of euthanasia, doctor-assisted killing, and suicide within the context of contemporary Chinese society. It has shed light on the intricate interplay between legal ambiguity, cultural norms, ethical considerations, and the psychological dimensions of suicide among acutely-ill patients. In doing so, this research has contributed significantly to the evolving discourse on bioethics and end-of-life decision-making in China.

At the heart of this discourse is the ongoing debate surrounding the legality of euthanasia and doctor-assisted killing. The absence of clear legislation in China has created a legal gray area, reflecting a profound conflict between traditional cultural beliefs and modern ethical considerations. This legal ambiguity not only poses challenges for healthcare professionals and patients but also impacts societal attitudes toward life-ending decisions. The findings of this study highlight the necessity for more comprehensive legal frameworks that can balance ethical principles with cultural sensibilities and the realities of medical practice.

Furthermore, the psychological aspects of suicide rumination among acutely-ill patients, as revealed in this study, underscore the complex relationship between personal suffering, mental health, and societal expectations. The significant role of factors such as familial burden, loss of dignity, and cognitive depression in influencing patients' acceptance of suicide is a stark reminder of the need for a more empathetic and nuanced approach to end-of-life care. This approach must integrate psychological support, ethical considerations, and respect for patient autonomy.

Moreover, the study's insights into the cultural and philosophical underpinnings of attitudes toward euthanasia and suicide in China are particularly noteworthy. The influence of Confucianism, Buddhism, and Taoism underscores the importance of understanding cultural contexts in bioethical discussions. As global bioethics continue to evolve, it is imperative to recognize and respect these cultural nuances, particularly in a country as diverse and historically rich as China.

In conclusion, this research provides a valuable contribution to the understanding of the ethical dilemmas and legal ambiguities surrounding euthanasia, doctor-assisted killing, and suicide in China. It underscores the need for a more integrated approach that considers legal, cultural, ethical, and psychological dimensions in addressing the challenges of end-of-life decision-making. As the global conversation on bioethics progresses, this study serves as a crucial reference point for policymakers, healthcare professionals, and scholars in navigating the complex terrain of life-ending decisions in a rapidly changing world.

## Data availability statement

The raw data supporting the conclusions of this article will be made available by the authors, without undue reservation.

## Ethics statement

Ethical review and approval was not required for this study involving human participants in accordance with the national legislation and institutional requirements. The studies were conducted in accordance with the local legislation and institutional requirements. The participants provided their written informed consent to participate in this study.

## Author contributions

GL: Conceptualization, Resources, Formal analysis, Funding acquisition, Methodology, Software, Writing – original draft, Writing – review & editing. KL: Conceptualization, Data curation, Formal analysis, Investigation, Methodology, Project administration, Writing – original draft, Writing – review & editing.
